# Role of cone-beam computed tomography (CBCT) in diagnosis and treatment planning of two-rooted maxillary lateral incisor with palatogingival groove. Case report

**DOI:** 10.4317/jced.57092

**Published:** 2020-07-01

**Authors:** Teresa Giner-Lluesma, Pedro Micó-Muñoz, Ilaria Prada, Pablo Micó-Martínez, Nicolas Collado-Castellanos, Alberto Manzano-Saiz, Alberto Albero-Monteagudo

**Affiliations:** 1 Prof. Asociado de Endodoncia y Odontología Restauradora, Universidad Europea de Valencia, España; 2 Prof. Titular de Endodoncia y Odontología Restauradora, Universidad Europea de Valencia, España; 3 Graduada en Odontología por la Universidad Europea de Valencia, Master Universitario en Odontopediatría Universidad Católica de Valencia, España; 4 Graduado en Odontología por la Universidad Europea de Valencia. Máster en Periodoncia y Osteintegración por la Universidad de Valencia, España

## Abstract

**Background:**

The embryonic root groove is an anatomical abnormality that starts in the cingulum and extends longitudinally down the long axis root towards the apex. This developmental anomaly is more frequently reported in maxillary lateral incisors. Gu YC in 2011 established three types of radicular grooves depending on its severity. According to this classification, type III presents a greater diagnostic and therapeutic complexity. The prevalence of palatogingival grooves in maxillary lateral incisors ranges from 1.9 to 14%. This case report provides valuable information about the diagnosis and treatment plan of palatogingival grooves with Cone-beam computed tomography (CBCT) scan.

**Case Report:**

The patient was referred to the University Dental Clinic of European University of Valencia, with recurrent abscesses at the upper right lateral incisor region for the last two years. Palpation and percussion tests were positive for tooth 1.2. There was no clinical history of caries or previous trauma. Periapical radiography showed periapical radiolucent lesions located, not only in the apical area of tooth 1.2, but also in tooth 1.3. Both teeth had previously been endodontically treated. Periodontal probing showed normal values. CBCT scan was perfomed in order to establish a definitive diagnosis and appropriate treatment plan.

**Discussion:**

The complex anatomy of the palatal root groove requires detailed knowledge of the internal root morphology for endodontic treatment success. This complementary tool allows a more accurate image of hard tissue structures, such as palatal grooves and/or accessory roots, in comparison to conventional periapical radiography. The treatment plan of this primary periodontal lesion with secondary endodontic involvement was as follows: periapical surgery combined with root amputation and sealing with MTA, and guided bone regeneration.

** Key words:**Palatal radicular groove, palatogingival groove, maxillary lateral incisor, cone-beam computed tomography, endodontic-periodontal lesion, guided bone regeneration.

## Introduction

The embryonic root groove is a developmental anomaly in which an invagination or fissure that starts in the cingulum, extends variably down the long axis root towards the apex (1). Withers *et al.* (2) observed that palatogingival grooves are found in 2.3% of upper incisors (4.4% of lateral incisors and 0.28% of central incisors). The incidence is totally variable (2.8% - 18%) depending on race, gender and employed diagnostic criteria (1-3).

While the cause of this anomaly is still not known, some authors believe it could be a type of “dens invaginatus”, a rare dental malformation caused by an infolding of enamel organ and Hertwig epithelial root sheath, during odontogenesis (4).

Gu Y-Ch (2011) (5) classified the embryonic root groove in 3 categories according to location, length, depth, complexity and severity. Type III are considered the greatest diagnostic and therapeutic challenge, being able to present C-shaped root canal conFiguration, root invagination and accessory mesial or distal root canal.

Although the main etiology of periodontal diseases is dental plaque, there are local factors such as embryonic root grooves that enhance periodontal attachment loss and hinders dental plaque removal by current oral hygiene measures, thus contribute to disease progression. Furthermore, this primary periodontal lesion may affect the dental pulp and cause endodontic-periodontal lesions or retrograde pulpitis. (6,7).

Intraoral periapical radiographs are useful complementary diagnostic tests, but their bidimensionality makes these embryonic root grooves difficult to diagnose. Therefore, more precise diagnostic tools, as the CBCT scan, are required to obtain reconstructed three-dimensional images of teeth and surrounding tissues (8,9).

The treatment choice will depend on the severity of these endo-periodontal lesions, but the root canal treatment on its own will generally not be effective (10). The prognosis of these teeth will depend on the extension of the periodontal lesion and the depth and accessibility of the groove (11).

This case report provides valuable information about the diagnosis and treatment plan of palatogingival grooves with Cone-beam computed tomography (CBCT) scan.

## Case Report

A 25-year old healthy Spanish female attended to the University Dental Clinic of European University of Valencia with recurrent abscesses at the upper right lateral incisor region for the last two years. The patient did not report any previous traumatism and clinically, no caries was observed. Nevertheless, adhesive restorations were found on the palatal side of the teeth 1.2 and 1.3. These teeth were not mobile but were sensitive to percussion and palpation, especially tooth 1.2. Furthermore, thermal and electrical pulp vitality tests were negative. Periodontal probing was performed after local anesthesia, and no periodontal pockets were found.

**Figure 1:**
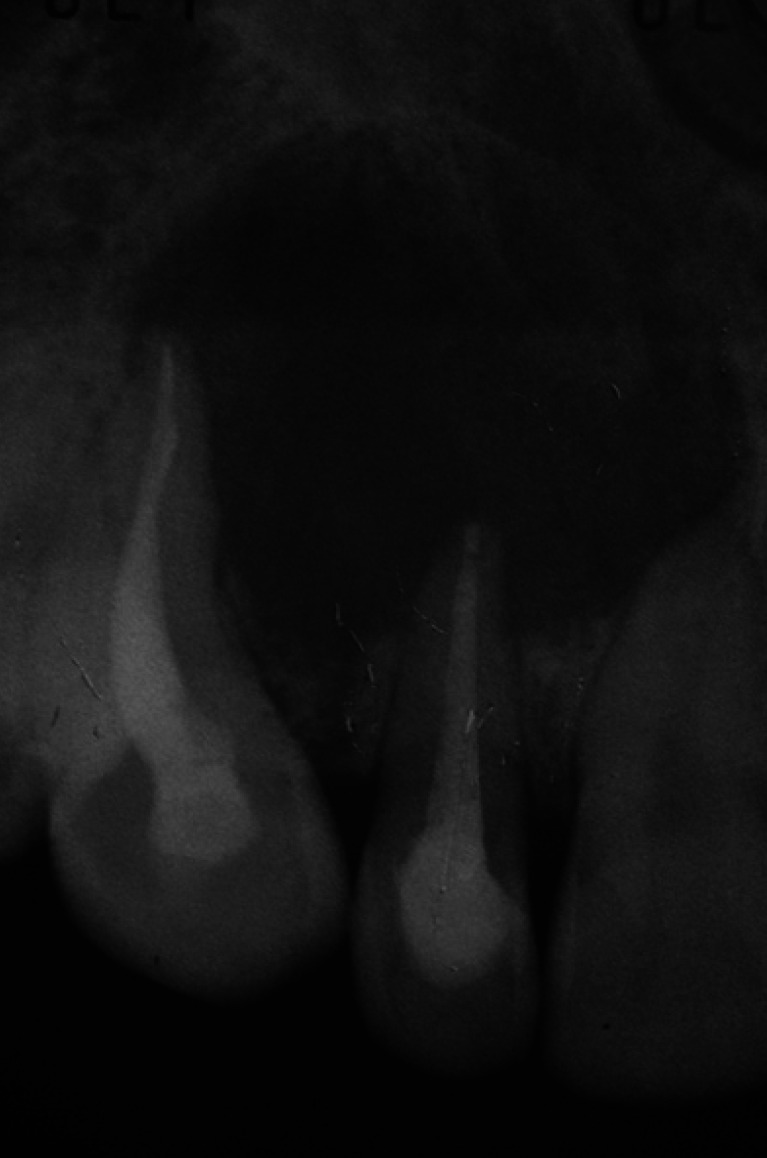
2.1 Intraoral periapical radiography. Big periapical radiolucency involving endodontically treated canine and right lateral incisor apex.

Periapical radiographs were taken from different projection angles. A large radiolucent area (>1cm) was observed around the apex of the endodontically treated teeth 1.2 and 1.3 (Fig. 1).

**Figure 2:**
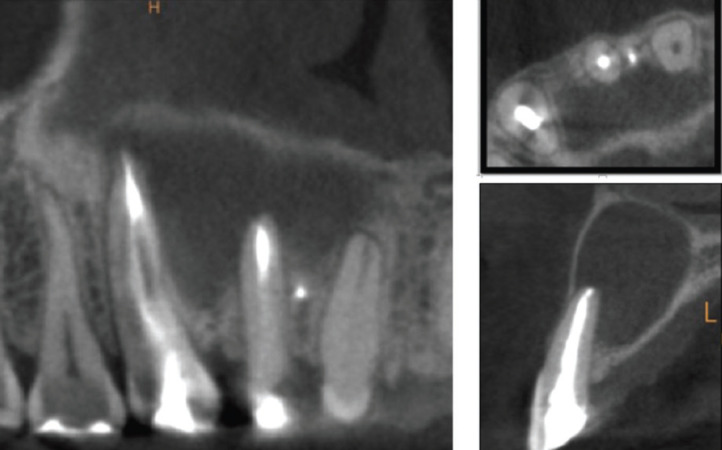
CBCT Coronal, axial and sagittal views of superior right lateral incisor with accessory root presenting a big periapical radiolucency.

**Figure 3:**
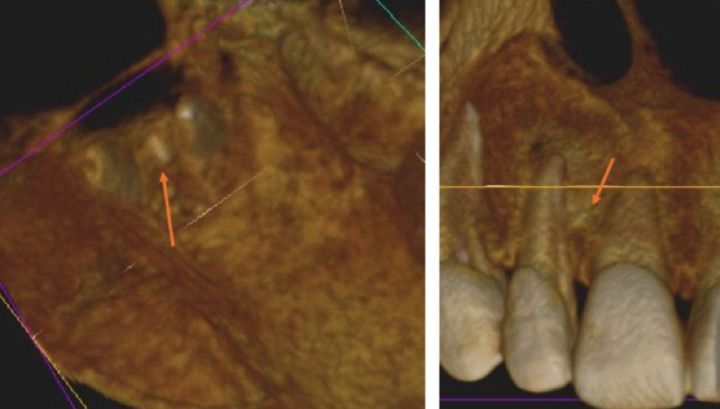
3D CBCT view of superior right lateral incisor with accessory root.

After conducting the conventional clinical and radiological assessment, a CBCT scan (Kodac CS 9000 Carestream ®) was performed to establish a more accurate diagnosis and treatment plan. CBCT images revealed, in the middle third of tooth 1.2, the presence of a non-endodontically treated accessory root (Fig. 2). Furthermore, the axial and coronal planes showed a radiopaque image in this accessory root, probably derived from the sealer cement of the main root canal (Fig. 2). In addition, the 3D reconstruction showed the existence of a second apex, belonging to the accessory root (Fig. 3).

Based on clinical and radiological findings, an endo-periodontal lesion associated to a palatogingival groove (type III according to Gu Y-Ch (5)) found in the upper right lateral incisor was considered the definitive diagnosis. The treatment plan of this primary periodontal lesion with secondary endodontic involvement was as follows: periapical surgery combined with root amputation and sealing with MTA, and guided bone regeneration for reconstruction of bone defect.

## Discussion

This article describes, with the help of CBCT scan, the diagnosis and treatment planning of a class III embryonic root groove with an accessory root in an endodontically-treated upper right lateral incisor. This tooth presented a severe endo-periodontal complication that encompassed the apex of teeth 1.2 and 1.3.

Palatogingival grooves can cause pulpal and periodontal pathologies, but they may be difficult to be identified as an etiological factor. The identification of these lesions can be clinically and radiologically challenging in early stages, especially if an acute abscess does not occur. Furthermore, root grooves or accessory roots can sometimes be mistaken for vertical root fractures (8). In addition, the diagnosis of endo-perio lesions caused by developmental anomalies is not normally conclusive due to the presence of nonspecific symptoms. This is why it is often confused with a purely periodontal or endodontic lesion (12). The prognosis of these teeth is questionable and mainly depends on groove location, depth and extension (8,13).

The patient came to our department with a complaint of recurrent abscesses. After performing percussion and palpation tests, clinical examination revealed slight pain on both teeth 1.2 and 1.3. In addition, despite the vast majority of authors relate the presence of a deep and narrow pocket to palatogingival grooves (6-8,12,14,15), periodontal pockets were not found in this patient after probing under local anesthesia, probably due to the fistula reorganization. In addition, both teeth 1.2 and 1.3 had been endodontically treated, but still presented a large radiolucent area that encompassed both apex. All of these conditions made the diagnosis even more challenging.

Radiographic examination is vital to detect embryionic grooves. Nevertheless, sometimes two-dimensional x-rays may not be sufficient to observe and understand the complex root system of these teeth. Some authors (8,11,12) affirm that a thin parapulpal radiolucent line can be observed in x-rays of teeth with palatogingival grooves, even in endodontically treated teeth. In this case, this parapulpal line was not observed in the two-dimensional x-ray, however, a large apical radiolucency encompassing both endodontically treated 1.2 and 1.3 roots was detected. A CBCT scan (Kodac CS 9000 Carestream) was taken to precisely visualize the apical lesion, to comprehend the etiology of this pathology, and to decide the treatment plan based on the findings.

Castelo-Baz *et al*. (2015) (13) confirm that the palatogingival groove presents a difficult diagnostic and treatment challenge, however many limitations of the conventional radiographs can be overcome by using the CBCT scan. Three-dimensional images are useful for assessment of spatial relationships between lesions and surrounding anatomical structures. In this case the three reference planes offered us the possibility to evaluate the root dimensions, morphologies and divisions, as well as possible accessory canals (12). Tan X. *et al*. (11) concluded that the axial CBCT images allow the best visualization of the grooves depth and permit to classify the embryonic grooves according to different anatomic variations. Therefore, the axial plane provides useful information about possible treatment complications. In this case report, the CBCT images were crucial to establish the diagnosis of endo-periodontal lesion affecting the upper right lateral incisor with an accessory root, caused by a palatogingival groove type III, according to Gu Y-Ch (2011) classification (5).

There are different treatment options available for endo-periodontal lesions, non-surgical and surgical procedures (12). In this case, we did not localize deep periodontal pockets and the endodontic treatment seemed radiographically correct, this is why we directly proposed the following surgery: accessory root amputation, granulation tissue elimination, application of mineral trioxide aggregate (MTA) in the apical portion of the root and guided bone regeneration for restoration of the original ridge volume.

Dentists should consider the possible anatomical variations of upper lateral incisors, carefully examine the preoperative radiographs, and make use of the CBCT as an additional diagnostic tool. Thus, the CBCT shows us more precisely the possible existence of the palatogingival groove and/or accessory root. An early diagnosis can increase the treatment success rate, however, the long-term prognosis of these lesions remains uncertain.
